# Book Reviews

**Published:** 1904-02

**Authors:** 


### BOOK REVIEWS.

THE BLUES (SPLANCHNIC NEURASTHENIA), CAUSES AND CURE. By Albert Abrams, A.M., M.D. (Heidelberg), F.R.M.S., Consulting Physician, Denver National Hospital for Consumptives; the Mount Zion and the French Hospitals, San Francisco; President of the Emanuel Sisterhood Polyclinic; formerly Professor of Pathology and Director of the Medical Clinic, Cooper Medical College, San Francisco.

The author will be remembered in connection of the humorous "Transactions of the Antiseptic Club," which appeared some years ago and which forms a contrast to his present work "The Blues."

This book concerns a variety of neurasthenia which, according to the author, is characterized by fits of depression of varying duration, and has its origin in a congestion of the intra-abdominal veins. The author regards this form of neurasthenia as quite amenable to treatment. The treatment is based upon physiological reasoning, and principally consists in producing a better tone to the abdominal muscles. This theory is elaborated in a volume of 225 pages.

---

A NON-SURGICAL TREATISE ON DISEASES OF THE PROSTATE GLAND AND ADNEXA. By George Whitfield Overall, A.B., M.D., Late Professor Physiology, Memphis Hospital Medical College, Chicago, Ill. Rowe Publishing Co., Chicago, Ill.

This book deals with an organ and neighboring structures which are especially prone to disease, and of which the treatment is very unsatisfactory. The author's success as detailed in the narrative of typical examples of the various diseased conditions encountered in this part of the genito-urinary tract would incline one to become an enthusiastic electro-therapeutic (for electro-therapeutics constitute the bulk of the remedial measures suggested).

One is likely to be arrested at the threshold of his enthusiasm by two important circumstances: the cost of a proper equipment and the admonition that none but the skilled in electric technique should essay to treat disease by this means. These two objections are hard to overcome and will doubtless circumscribe the work to a few as heretofore.

The book deals with the anatomy of the prostate, inflammation of it, acute and chronic, hypertrophy of the prostate, neuroses having their origin in the prostate, and a chapter on affections of seminal vessicles.

The book is well illustrated, the language used in the descriptions good, and the directions for following out the several plans of treatment clear.

---

### AMERICAN DENTISTS TO ROYALTY.

One of Emperor William's best friends is his American dentist, Dr. Sylvester, who lives in a magnificent mansion near the Thiergarten in Berlin. When the Kaiser wants his teeth attended to he never sends for Dr. Sylvester to come to him, but goes himself to the dentist's house on foot. The Emperor has loaded the doctor down with gifts and favors.

With the King of Saxony Dr. Jenkins has always enjoyed very great favor. The King will never have any other dentist, and this partially has made Dr. Jenkins enormously rich.

Dr. Wallason, the Czar's American dentist, lives in St. Petersburg in a palace in the quarter reserved for Grand Dukes and Ambassadors. Probably no one living knows the imperial family as intimately as Dr. Wallason.

Dr. Thomas, an American dentist of Vienna, has been for years an intimate friend of the Emperor.

---

### BLAND TINCTURE OF IODINE.

Claret (*Journal de Pharm. et de Chemie*) is authority for the statement that the addition of two parts of borax (sodii biborati) to one part of tincture of iodine renders the iodine solution stable and non-irritating. The formation of hydriodic acid, which gives to the tincture its irritant properties, is at once taken up by the liberation of boric acid, an indifferent substance.

